# Risks and cancer associations of metachronous and synchronous multiple primary cancers: a 25-year retrospective study

**DOI:** 10.1186/s12885-021-08766-9

**Published:** 2021-09-23

**Authors:** Pariyada Tanjak, Bhoom Suktitipat, Nutchavadee Vorasan, Panudeth Juengwiwattanakitti, Benjarat Thiengtrong, Cholticha Songjang, Suwanit Therasakvichya, Somsri Laiteerapong, Vitoon Chinswangwatanakul

**Affiliations:** 1 Siriraj Cancer Center, Faculty of Medicine Siriraj Hospital, Mahidol University, 2 Wanglang Road, Bangkok Noi, Bangkok, 10700 Thailand; 2 Department of Surgery, Faculty of Medicine Siriraj Hospital, Mahidol University, Bangkok, Thailand; 3 Department of Biochemistry, Faculty of Medicine Siriraj Hospital, Mahidol University, Bangkok, Thailand; 4 Siriraj Center of Excellent for Research in Bioinformatics and Clinical Data Management, Faculty of Medicine Siriraj Hospital, Mahidol University, Bangkok, Thailand; 5 Integrative Computational Bioscience Center, Mahidol University, Nakhon Pathom, Thailand; 6 Siriraj Genomics, Faculty of Medicine Siriraj Hospital, Mahidol University, Bangkok, Thailand; 7 Department of Gynecology, Faculty of Medicine Siriraj Hospital, Mahidol University, Bangkok, Thailand; 8 Department of Transfusion Medicine, Faculty of Medicine Siriraj Hospital, Mahidol University, Bangkok, Thailand

**Keywords:** Cancer registry, Metachronous, Multiple primary cancers, Second primary cancer, Synchronous

## Abstract

**Background:**

The situation of patients developing multiple primary cancers is becoming more frequent and graver. This study investigated the risks of developing second primary cancers that are related to first primary cancers, and the interval times of synchronous and metachronous multiple primary cancers.

**Patients and methods:**

Retrospective data were retrieved from 109,054 patients aged ≥18 who were diagnosed with a first solid cancer and registered at Siriraj Cancer Center between 1991 and 2015. A two-month period between first- and second- primary cancers was used to differentiate metachronous and synchronous multiple primary cancers. The combinations of subsequent cancers and relative risks (RRs) of having multiple primary cancers versus having single primary cancer for the top-ten first and second primary cancers were examined. The RR was adjusted for age of the first primary cancer. A survival analysis of the time to second-primary-cancer development was performed.

**Results:**

Multiple primary cancers were found in 1785 (1.63%) patients. Most (70.87%) second primary cancers occurred after 2 months of first breast, skin, colorectal, lung, head and neck, liver, male genital cancer–prostate, thyroid, and female genital cancer–non-uterine cancers, resulting in those cancers being classified as metachronous multiple primary cancer. After adjustment for age at first diagnosis, head and neck cancers had the highest metachronous association with second esophageal cancers (RR, 25.06; 95% CI, 13.41–50.77). Prostate cancer and second colorectal cancer also demonstrated a high metachronous association (RR, 2.00; 95% CI, 1.25–3.05). A strong synchronous association was found between uterine and ovarian cancers (RR, 27.77; 95% CI, 17.97–43.63). The median time from the first uterine cancer to second-cancer development was 55 days.

**Conclusions:**

The top-ten most frequent multiple primary cancers were the following: breast; liver; head and neck; colorectal; male genital cancer–prostate; skin; female genital cancer–uterine; thyroid; lung; and female genital cancer–non-uterine. Second primary cancers showed specific associations that depended on the first primary cancer. Physicians should be cognizant of the most common combinations and the interval times of metachronous and synchronous multiple primary cancers.

## Background

The survival rate of cancer patients is improving globally. In addition to steady advances in diagnostic techniques and treatment modalities, early detection and proper management are contributing to the prolonging of the survival of cancer patients [1–3]. Patients with a history of cancer may develop several cancers in their lives [4]. When a patient is diagnosed with more than one cancer, multiple primary cancers may be reported. Recently, the prevalence of multiple primary cancers has risen [5–7]. A better understanding of the characteristics and risks of multiple primary cancers may be beneficial for cancer management.

The term “multiple primary cancers” refers to the synchronous or metachronous appearances of cancers in the same individual, but does not include instances of the metastasis of initial primary cancers [8]. The Surveillance, Epidemiology, and End Results (SEER) Program recommended that second primary cancers that occurred within 2 months of the first primary tumor should be defined as synchronous multiple primary cancers [9, 10]. The presence of synchronous and metachronous malignant cancers is problematic if each of the cancers is active [8]. The challenge is to find an anticancer therapy that addresses the active malignancies in each patient but does not increase toxicity, create undesirable pharmacological interactions, nor have an adverse impact on the overall outcomes [8]. Therefore, the prediction and prevention of second cancer occurrences may help to improve the quality of life and prolong overall survival of cancer patients [11].

Retaining a cancer registry is one of the most effective approaches to studying cancer epidemiology and healthcare-management planning worldwide. Siriraj Hospital, Mahidol University, is the largest tertiary hospital in Thailand, provide 2 million of outpatient services each year. The Siriraj Cancer Center was established in 1945. The performance of its cancer registry is regularly audited and reported in accordance with specifications prescribed in the World Health Organization–International Classification of Diseases for Oncology, 3rd Edition (ICD-O-3) [12]. The cancer registry houses accurate and essential hospital-based data of all patients who receive cancer treatment at Siriraj Hospital [13].

In addition to investigating the risks of solid multiple primary cancers in terms of the first and second primary cancers, we also aimed to estimate the time to the development of second cancers. We classified metachronous and synchronous multiple primary cancers using a cutoff of 2 months, in accordance with SEER criteria [9, 10]. Essentially, cancers occurring within 2 months of each other are deemed synchronous, whereas those developing later than 2 months are regarded as metachronous [9]. As the Universal Health Coverage system achieves in Thailand that more than 90% of the population can access healthcare systems [14], the survival of some cancer trends to increase [15]. Although, some site-specific cancer care survivorship guidelines were existed [16, 17], the development of appropriate surveillance strategies should be studied for improving the quality of life in Thai cancer population. The significance of this study lies in better understanding of types of secondary primary specific cancers and the durations until their occurrence. At a minimum, it should facilitate such knowledge that may assist with the development of appropriate long-term surveillance strategies.

## Methods

### Ethical approval and data set

Before commencement of the study, ethics approval was obtained from the Institutional Review Board of Siriraj Hospital (Si293/2018). Retrospective data were retrieved from the cancer registry of Siriraj Cancer Center, Faculty of Medicine Siriraj Hospital, Mahidol University [18]. The methods were performed in accordance with all approved guidelines.

### Patient cohort

Siriraj Cancer Registry was established in 1969 to track the prevalence of cancer at Siriraj Hospital. All patients diagnosed with new cancer or referred for first treatment were included in the registry. Validating data of staging, treatment and survival were periodically appended every 2–3 months. The endpoint of follow-up was death or loss of follow-up. However, a systematic and complete linkage of incidence cases against mortality data was not carried out for some patients. The initial cohort of 129,514 individuals included all new patients aged above 18 years who had been diagnosed with first primary cancers and registered with the Siriraj Cancer Center between 1991 and 2015.

The primary cancer site was defined as per the ICD-O-3 description [12], and it was categorized into 20 major types of solid cancer, based on topography codes. The types were breast, head and neck, liver, colorectal, lung, thyroid, ovarian, skin, bladder, stomach, brain and other nervous, esophageal, pancreatic, renal, bone and cartilage, heart and thymus, adrenocortical, female genital, male genital, and other cancers (sarcoma). Cancers of the female genital organ were classified as uterine and non-uterine, while cancers of the male genital organ were classified as prostate and non-prostate. Excluded were patients with 1842 malignant lymphomas; 11,780 cancer sites in the hematopoietic system, reticuloendothelial system, or lymph nodes; and 5061 unknown primary sites. 10 men with breast cancer were also not included. The resulting number of subjects in this study was 109,054.

**Fig. 1:**
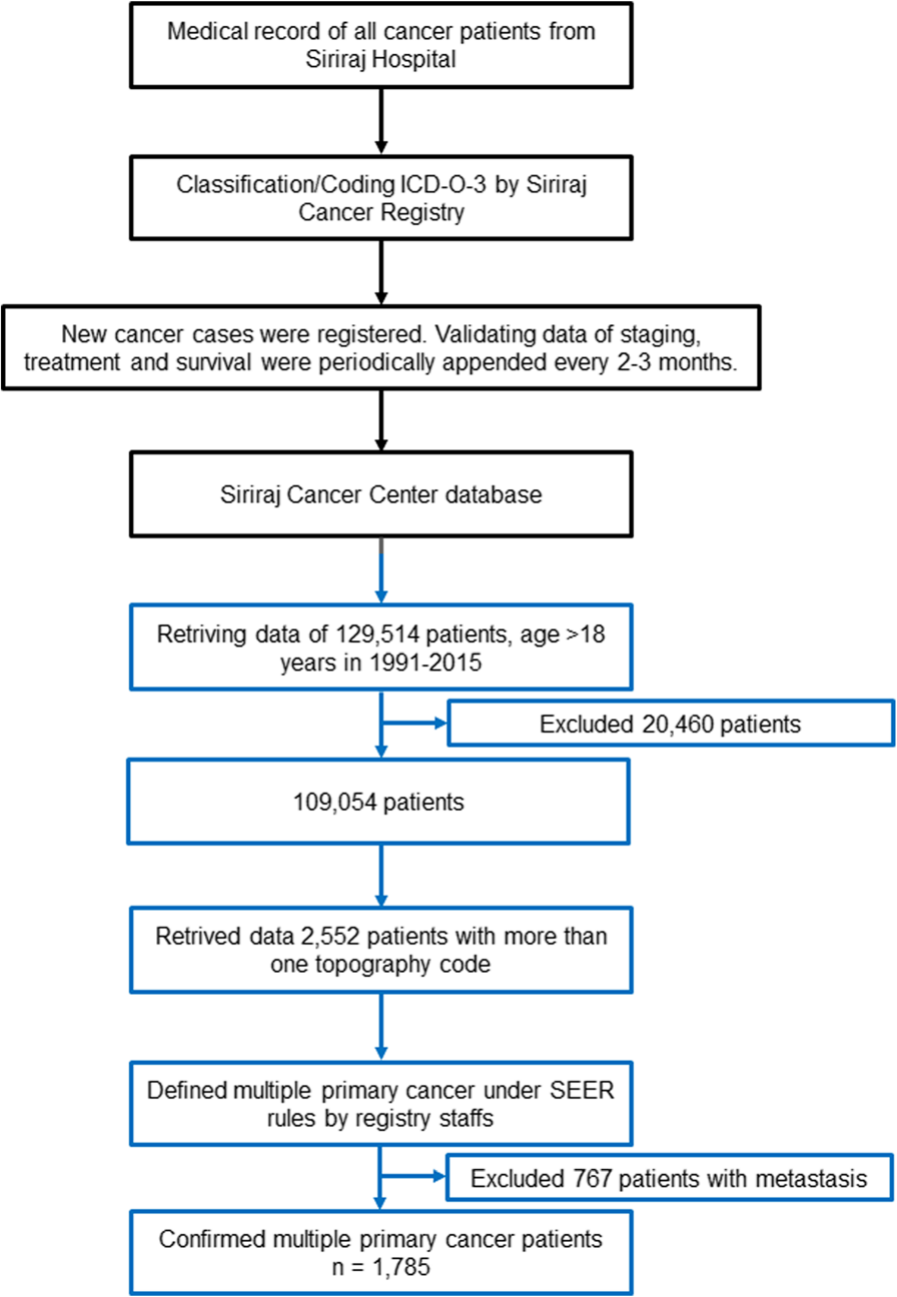
Flowchart of Siriraj Cancer Registry and eligible study cohort. Siriraj Cancer Registry (black line), eligible study cohort (blue line)

The final study cohort included 2552 patients who had more than one topography code allowing for a minimum of 2 months after first cancer diagnosis to determine the presence of second primary cancer. The patients with less than 2 months of follow-up were excluded from the analysis. The individual data such as date of diagnosis, histology, treatment, laterality of paired organ, of each patient in final study cohort were verified and clarified by registry staffs of the hospital. The multiple primary cancers were handled and identified by registry staffs using SEER rules [9]. Excluded were patients with 767 metastasis and recurrence of cancers (Fig. 1). With the regular data validating every 2–3 months, the framework of the Siriraj Cancer Registry effectively identify the time to the development of the second primary cancers.

### Definition of multiple primary tumors

This study utilized the definitions of metachronous and synchronous multiple primary tumors determined by the SEER Program of the National Cancer Institute [9]. Under the SEER rules, the term “metachronous multiple primary tumors” refers to cases in which second primary tumors are diagnosed more than 2 months after the diagnosis of the related first primary tumors [9, 10]. By comparison, “synchronous multiple primary tumors” are defined as second primary tumors occurring within 2 months of the first primary tumors [9, 10]. SEER rules also take into account of the topography, date of diagnosis, histology, and laterality of paired organs per lifetime [9]. In brief, the duration between the first and second cancer diagnoses, and second primary tumors whose histopathologies match those of their related first tumors, are considered. In addition, every segment of the colon is regarded as a single primary site [9].

### Statistical analysis

Analyses were performed using R (version 3.6; R Foundation for Statistical Computing, Vienna, Austria) [19]. The differences in clinical characteristics between the group with single primary cancer vs the group with multiple primary cancers were tested with chi-squared test for sex and t-test for age. Similarly, the differences in sex and age between metachronous and synchronous tumors were tested using chi-squared test and t-test, respectively. A *P* value of < 0.05 was considered a statistically significant difference. The relative risk (RR) for developing multiple primary cancers versus single primary cancer was estimated and adjusted for age of the first primary cancer using, quasi-poisson model via glm R package [19]. A survival analysis of the time to the development of the second primary cancers was performed using the “survminer” R package [20].

## Results

### The 25-year statistics of single and multiple primary cancers

We identified 109,054 patients who had been registered with a first solid cancer by the cancer registry of Siriraj Cancer Center over the 25-year period between 1991 and 2015. Of those cases, 1785 (1.63%) developed a second primary cancer (Table 1). Most cancer patients were female (57.3%). In addition, the patients with multiple primary cancers were significantly older than those with a single primary cancer (mean ages, 57.2 vs. 60.2 years; *P* <  0.001; RR, 1.01; 95% confidence interval [95% CI],1.01–1.02).

**Table 1:** Clinical characteristics of the analytical cohort. Clinical characteristics of patients with a single primary cancer and patients with multiple primary cancers

Clinical variable	Singleprimary cancer (***n*** = 107,269, 99.37%)	Multipleprimary cancers (***n*** = 1785, 1.63%)	Total (***n*** = 109,054)	RR (95% CI)	***P***
Sex				0.94 (0.85–1.03)	0.164
Male	45,834 (42.7%)	792 (44.4%)	46,626 (42.8%)		
Female	61,435 (57.3%)	993 (55.6%)	62,428 (57.2%)		
Age at 1st diagnosis				1.01 (1.01–1.02)	< 0.001
Mean (SD)	57.2 (14.517)	60.2 (13.2)	57.2 (14.5)		
Range	18.0–106.0	18.0–103.0	18.0–106.0		

Abbreviations: *95% CI* 95% confidence interval; *RR* relative risk of multiple primary cancers vs single primary cancer

**Fig. 2:**
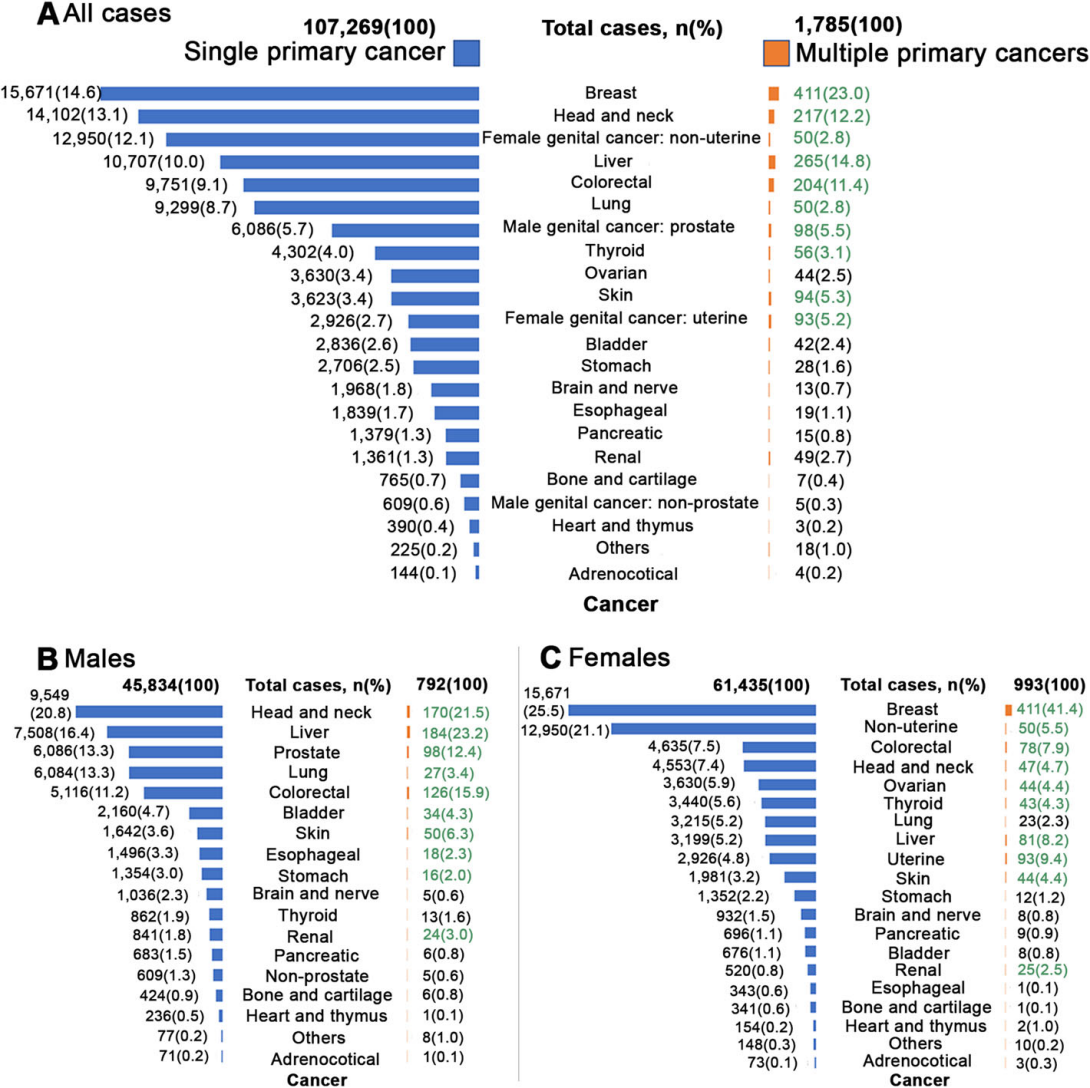
The distribution of single primary cancer and multiple primary cancers. The distribution of single primary cancer and multiple primary cancers of 109,054 patients from 1991 to 2015 for all cases (A), males (B), and females (C). The green items represent the top-ten, first-diagnosed cancers of metachronous and synchronous multiple primary cancers for all cases, males, and females

The 25-year, the numbers of patients of single and multiple primary cancers at Siriraj Hospital are illustrated in Fig. 2A. Breast cancer was the most frequent form of single primary cancer (14.6%) and multiple primary cancer (23%). The top-ten multiple primary cancer types were the following: breast (23.0%); liver (14.8%); head and neck (12.2%); colorectal (11.4%); male genital cancer–prostate (5.5%); skin (5.3%); female genital cancer–uterine (5.2%); thyroid (3.1%); lung (2.8%); and female genital cancer–non-uterine (2.8%). Nearly all of those types were also found in the top-ten single primary cancers. Figure 2B and C present the distributions of the single and multiple primary cancers in 792 (44.4%) males and 993 (55.6%) females. The most common cancers were gender-specific.

### The top-ten, first-diagnosed cancers of metachronous and synchronous multiple primary cancers

Of the 1785 patients with multiple primary cancers, there were 1265 (70.87%) cases of metachronous multiple primary cancers and 520 (29.13%) cases of synchronous multiple primary cancers (Fig. 2A). Most of the multiple primary cancer patients were female (55.6%), and there were statistical differences in the distributions of the metachronous and synchronous cancers of each gender group (*P* <  0.001). The mean age at diagnosis of the first metachronous and synchronous cancers was 60 years (Table 2).

**Table 2:** Clinical characteristics of multiple primary cancers patients. Clinical characteristics of patients with metachronous and synchronous multiple primary cancers

Clinical variable	Metachronous multiple primary cancers (***n*** = 1265, 70.87%)	Synchronous multiple primary cancers (***n*** = 520, 29.13%)	Total (***n*** = 1785)	***P***
Sex				< 0.001^a^
Male	596 (47.1%)	196 (37.7%)	792 (44.4%)	
Female	669 (52.9%)	324 (62.3%)	993 (55.6%)	
Age at 1st diagnosis				0.691^b^
Mean (SD)	60.2 (13.1)	60.0 (13.5)	60.1 (13.2)	
Range	18.0–96.0	20.0–103.0	18.0–103.0	

Abbreviations: ^a^ chi-squared test; ^b^ t-test

**Fig. 3:**
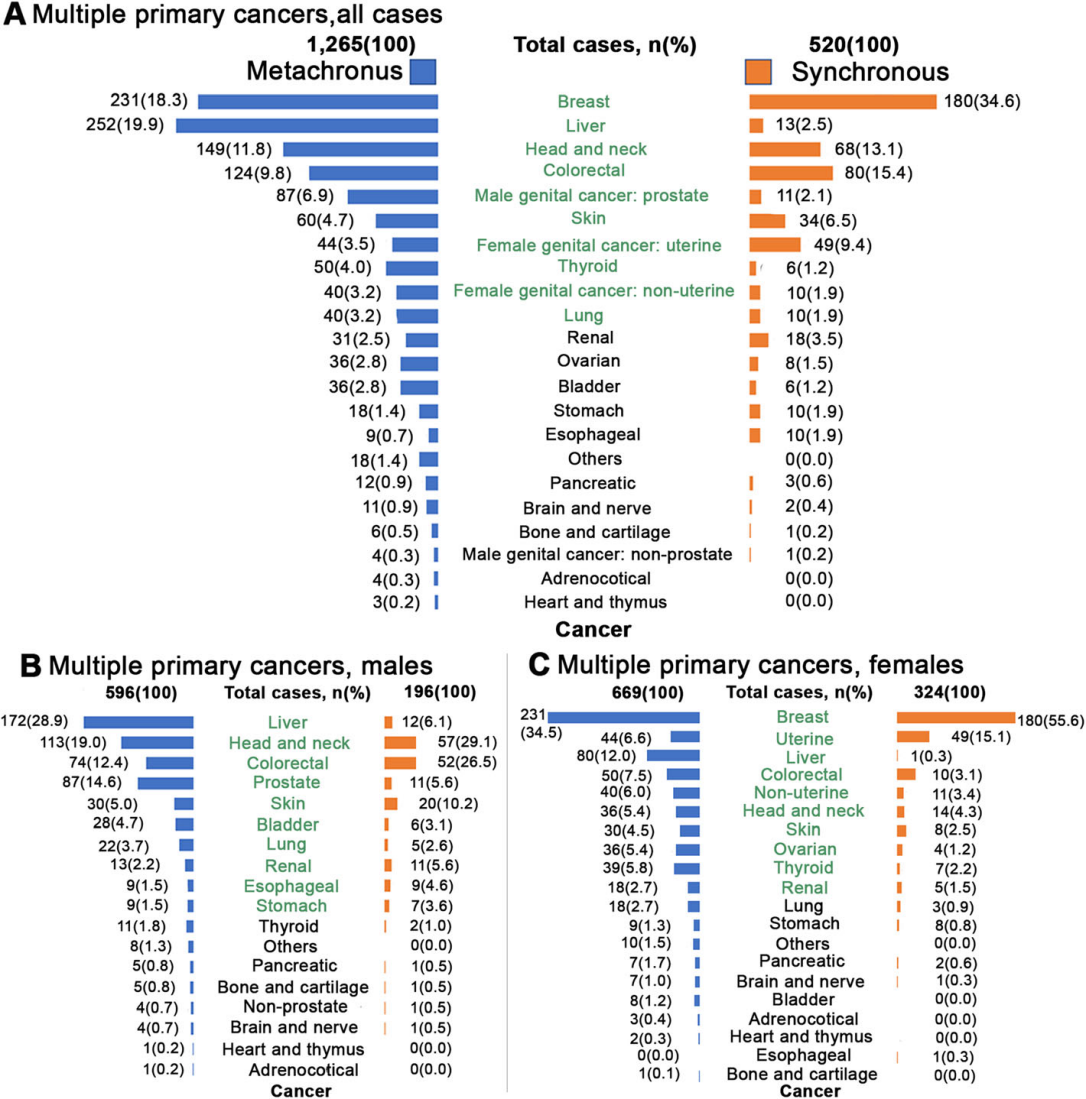
The distribution of the first-diagnosed cancers of multiple primary cancers. The distribution of the first-diagnosed cancers of metachronous and synchronous multiple primary cancers for all cases (A), males (B), and females (C). The green items represent the top-ten, first-diagnosed cancers of metachronous and synchronous multiple primary cancers for all cases, males, and females

Figure 3A, B, and C illustrate the distribution of metachronous and synchronous multiple primary cancers in all cases, males, and females, respectively. Most of the multiple primary cancers were metachronous multiple primary cancers (70.87%). We found that the survivors from breast cancer had the highest risk of developing metachronous (18.3%) and synchronous (34.6%) multiple primary cancers. The top-ten metachronous multiple primary cancer types were the following: liver (19.9%); breast (18.3%); head and neck (11.8%); colorectal (9.8%); male genital cancer–prostate (6.9%); skin (4.7%); thyroid (4.0%); female genital cancer–uterine (3.5%); lung (3.2%); and female genital cancer–non-uterine (3.2%). Female genital cancer–uterine had a higher incidence of synchronous (9.4%) than metachronous (3.5%) multiple primary cancers. It was also noted that most of the second primary cancers occurred more than 2 months after the first primary cancers had been diagnosed; the exception was female genital cancer–uterine, which tended to occur within 2 months.

### Times to second cancers

**Fig. 4:**
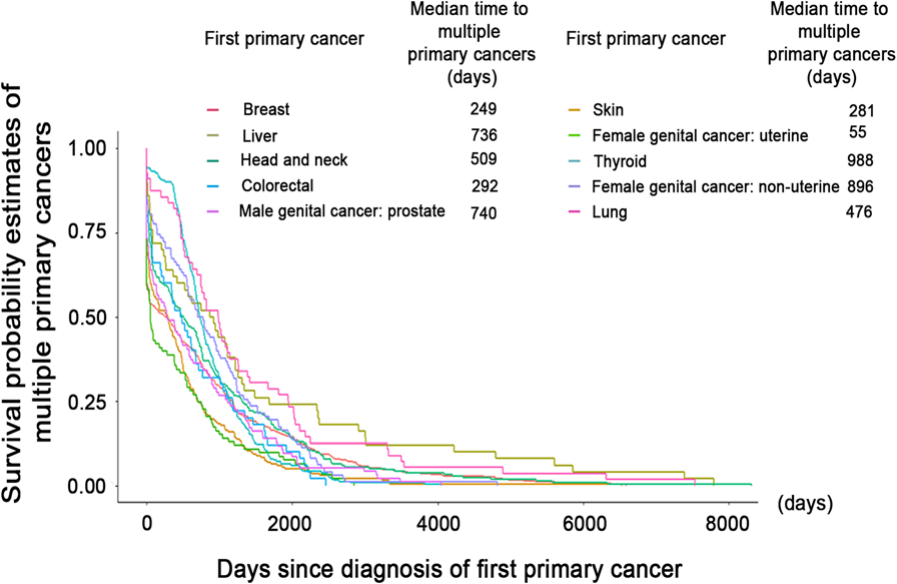
Time to develop multiple primary cancers. The median times to the development of multiple primary cancers, stratified by the first cancer that had developed in patients

Based on the results of the survival analysis, the median times to the development of the second cancers of the top-ten multiple primary cancers are illustrated in Fig. 4. The median times differed among the cancer types. The shortest duration (55 days) was between the diagnosis of first female genital cancer–uterine and the detection of a subsequent cancer. Next were the median times following breast, skin, and colorectal cancer diagnoses (each within 1 year). Second cancers developed within 2 years for first lung cancers and for head and neck cancers. Longer medians (more than 2 years) were revealed for the development of second cancers following diagnoses of liver, male genital cancer–prostate, thyroid, and female genital cancer–non-uterine.

### The combinations of first primary cancers and metachronous and synchronous second primary cancers

**Fig. 5:**
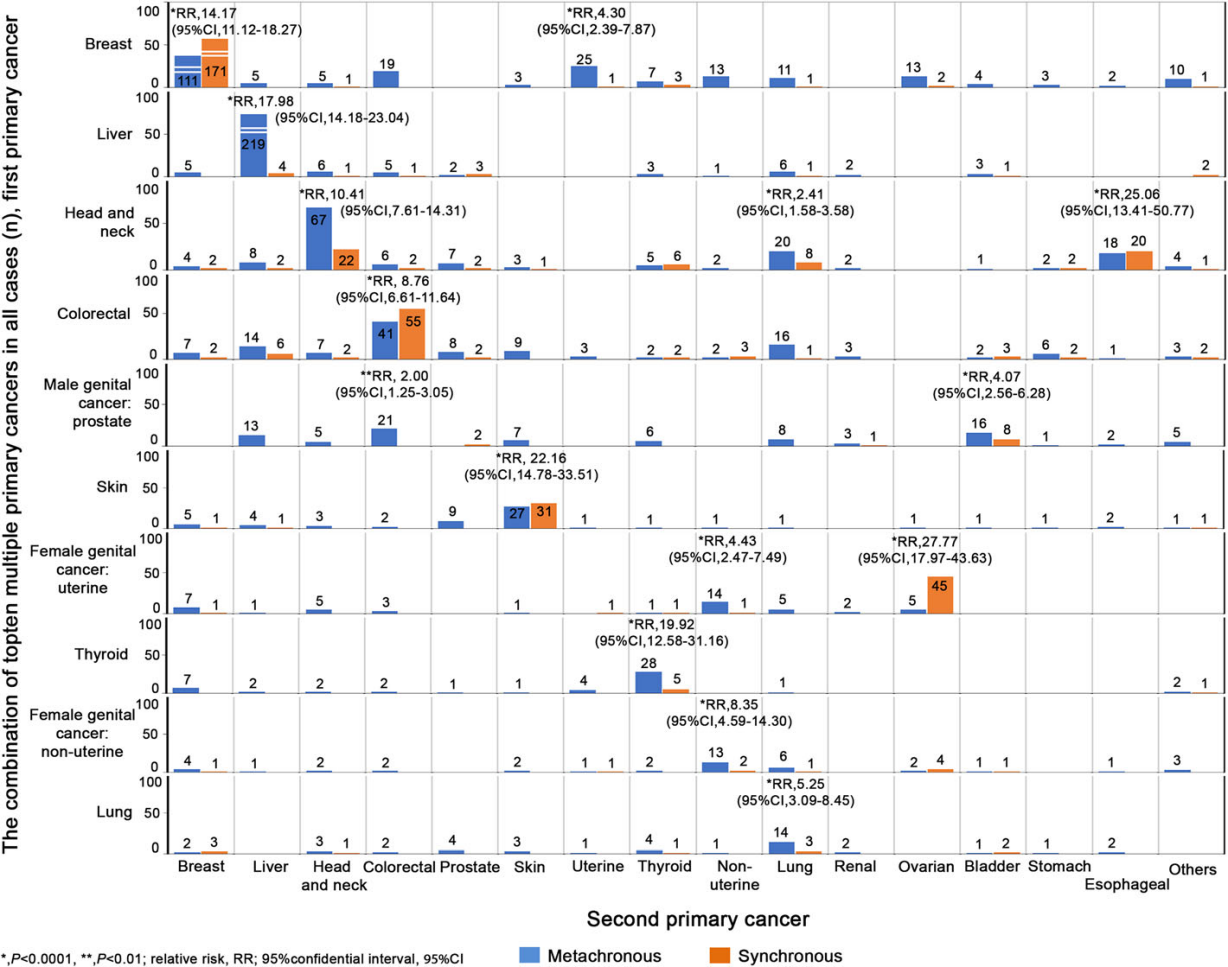
The risk of top-ten combination cancers. The combination cases of the top-ten first primary cancers and subsequent second primary cancers for metachronous and synchronous multiple primary cancers (*, *P* < 0.0001; **, *P* < 0.01; 95% CI, 95% confidence interval; RR, relative risk of having multiple primary cancer versus having single primary cancer, adjusted for age at first primary cancer)

From the 10 first multiple primary cancers of all cases, we predicted the RRs adjusted for age between the first and second primary cancers. We also identified the combinations of first primary cancers and metachronous and synchronous second primary cancers (Fig. 5). The combination of first breast cancer and second breast cancer was statistically significant, compared with the combination of first breast cancer and other cancer types (RR, 14.17; 95% CI, 11.12–18.27; *P* < 0.0001). Additionally, female patients with breast cancer had an elevated risk of occurrence of second female genital cancer–uterine (RR, 4.30; 95% CI, 2.39–7.87; *P* < 0.0001), which mostly occurred after 2 months. We observed that head and neck cancers had a strong association with second esophageal cancer (RR, 25.06; 95% CI, 13.41–50.77; *P* < 0.0001), second head and neck cancer (RR, 10.41; 95% CI, 7.61–14.31; *P* < 0.0001), and second lung cancer (RR, 2.41; 95% CI, 1.58–3.58; *P* < 0.0001). Moreover, we found that male patients with first prostate cancer had an elevated risk of second bladder cancer (RR, 4.07; 95% CI, 2.56–6.28; *P* < 0.0001) and second colorectal cancer (RR, 2.00; 95% CI, 1.25–3.05; *P* < 0.01), both of which mostly developed after more than 2 months.

## Discussion

The increase in the number of multiple primary cancers is challenging worldwide. Understanding the characteristic of metachronous and synchronous multiple primary cancers may facilitate the development of better management plans for patients. We found that the 10 most common multiple primary cancers were the following types: breast, liver, head and neck, colorectal, male genital cancer–prostate, skin, female genital cancer–uterine, thyroid, lung, and female genital cancer–non-uterine. Those cancers also had a high cumulative incidence at Siriraj Hospital from 1991 to 2015. This finding is consistent with the common cancer types in Thailand and the United States [21, 22]. The survivors from these cancers may face a second primary cancer in their lifetime. Moreover, our results suggested that most of the patients with multiple primary cancers were older at diagnosis of their first cancer than were those diagnosed with a single primary cancer. This may reflect the poor prognosis for young adults with cancers, such as breast cancer [23] and colorectal cancer [24]. Breast cancers in young adults have a higher incidence of the more aggressive triple-negative form and are less hormone-sensitive than those in older adults [25]. As to colorectal cancers, they have a more mucinous histology and greater frequency of signet ring cells in young adults than in older adults, resulting in a markedly poor prognosis [26].

Among the patients with multiple primary cancers, there was a higher incidence of second cancers in females than males, which differs from the reported pattern of development of second cancers in the United States [11]. In the current study, we defined multiple primary cancers according to the SEER rules. The SEER Program considers topography, date of diagnosis, histology, and laterality of paired organs per lifetime [9]. Under the SEER rules, second primary breast cancers whose histopathologies match those of the related first breast cancers, and the duration to cancer diagnosis, are considered [9]. Moreover, each segment of the colon is regarded as a single primary cancer [9]. However, these rules differ from those used for multiple primary cancers by the World Health Organization’s International Agency for Research on Cancer (IARC) and the International Association of Cancer Registries (IACR) [27, 28]. The IARC/IACR does not define multiple primary cancers depending on time; instead, only one tumor (depending on the histological group) per organ or pair of organs per person per lifetime is reported [27, 28]. Furthermore, the significantly high levels of breast cancer in all regions of Thailand [21] may have increased the number of multiple primary cancers that the present study found among females.

Breast cancer, the most frequently found multiple primary cancer, was commonly associated with second breast cancer and second female genital cancer–uterine. This is consistent with reports from the United States [29, 30]. The common combination of first breast cancer and second breast cancer may be explained by the increased expression of estrogen and progesterone receptors in patients with first primary breast cancers, thereby triggering the development of multiple primary cancers [31]. Similarly, the combination of first thyroid cancer and second thyroid cancer may be triggered by the increased levels of thyroid globulin and thyroid peroxidase in patients [31].

The metachronous and synchronous multiple primary cancers were differentiated by using the cutoff period of 2 months. We observed that most of the multiple primary cancers were metachronous. Only female genital cancer–uterine was assessed as having more synchronous than metachronous multiple primary cancers. Our results suggest that patients with uterine cancer should be monitored for the development of a second ovarian cancer within 2 months (median period, 55 days). The uterine and ovarian cancer association may be explained by either a mutation or an overexpression of *BRCA1/BRCA2*, and by *MMR* genes [32]. Patients with uterine cancer who have those genes should be immediately investigated for the presence of second primary tumors.

Head and neck cancers were among the ten most common multiple primary cancers. The combination of head and neck cancer was distributed among several cancer types within 2 years (namely, esophageal, head and neck, and lung), with high RRs after adjustment for age at first primary cancer. The development of the second primary head and neck cancers may result from radiation treatment [33].

Secondary colorectal cancer was found to be strongly associated with first male genital cancer–prostate and first colorectal cancer. Our results suggested that survivors of colorectal cancer should be monitored to detect second cancer development within 1 year. In contrast, patients with prostate cancer may develop a second cancer after longer than 2 years. The multiple primary tumors found in colorectal cancer may be associated with hereditary colorectal cancer and could be explained by genetic profiling. The occurrence of first colorectal cancer followed by second colorectal cancer have been commonly identified with *BRCA1/BRCA2*, and *MMR* genes [32]. The combination of prostate cancer and colon cancer share both genetic risk factors such as the *BRCA1/BRCA2* mutation [34] and dietary ones [35].

An improvement of diagnostic techniques and treatment modalities, early detection and proper management helps to prolong the survival of cancer patients [1–3]. Several site-specific cancer care survivorship guidelines were existed [16, 17]. Although those guidelines may be appropriate for survivors of some cancers, this current finding may incorporate with them to help estimate risk of a subsequent cancer. Our study suggested that multiple primary cancers may occur months or year after first cancers were diagnosed. Patients with first cancer diagnosis and survivors of some cancers may be screened for genetic risk factors such as *MMR* and *BRCA1/BRCA2* mutations [32, 34]. For example, patients with uterine cancer with those gene mutations should be immediately investigated for the presence of second primary tumors. Survivors with breast and colorectal cancer with genetic factors are advised to annual follow-up as guideline [17, 36], and our results suggest these patients should be followed within 1 year after first cancer diagnosis. During the follow-up, attention should be paid on the second primary cancers that showed specific association, and depended on the first primary cancer such as first breast and second breast cancer, first prostate and second bladder cancer, and first prostate and second colorectal cancer.

### Limitations

Although our study was based on data from population-based registry of Siriraj Hospital, the largest tertiary hospital in Thailand, the registry of one hospital is the limitation. As Thai government provides the majority healthcare through countrywide public hospitals including Siriraj Hospital, our study cases are mostly low- and middle-income patients as general population in public hospitals. However, Siriraj Hospital is one of the best-known medical school and the largest referral center of various complex diseases, patients from all regions of the country have access to the hospital. Therefore, the majority of cancer patients in this study may be more complexity of the disease than in general patient population.

Currently, mainly two definitions of multiple primary cancers are used, SEER [9] and IARC/IACR/ and European Network of Cancer Registries, ENCR [27]. For this study, we applied SEER rules in the definition of multiple primary cancers, would make it difficult to compare with IARC/IACR/ENCR. IARC/IACR/ENCR rules are less complex than the other leading to facilitate international comparison [37]. SEER rules may over count multiple primary cancers in the same anatomic site [37]. Whereas in separate organ systems, both rules present the similar count of multiple primary cancers [37]. This study lacked sensitivity analyses of both definition rules. However, a comparison of both definition rules was studied [37], and a combination of them was also reported [38].

This study adjusted for age, therefore improved aging itself does not increase risk estimates. The behavior such as smoking status [39] and sun protection [40] that are likely related to the development of the first and second primary cancers cannot be measured in this study. Patient demographics such as marital status, income, tumor grade and tumor stage were excluded in our analysis; however, another study found those confounding factors strongly associated with risks of a subsequent cancer [11]. The treatment types seem to influence the higher risk of the second primary head and neck cancers [33]. We were unable to collect information either family history of cancer or genetic risk factors that may identify high risk individuals.

Despite the competitive risk of survival between single and multiple primary cancers was not performed to analyze in this study. Another research found that patients with multiple primary cancer had longer survival than those with single primary cancer [41]; however, some cancer types such as multiple lung cancers indicated a poorer prognosis than single primary cancer [42].

## Conclusions

In conclusion, this study revealed evidence of a strong association between the co-occurrence of specific cancers in terms of metachronous and synchronous multiple primary cancers. Our results suggested that second primary cancers have a specific dependency on first primary cancers. Additionally, our results indicated that the risk of developing second cancers is probably time-dependent. Further studies on the genetic profiles of the associated primary cancers may expand our understanding of multiple primary cancers and thereby enhance cancer management.

## Data Availability

The datasets used and/or analysed during the current study are available from the corresponding author on reasonable request.
